# Ti–Si composite glycol salts: depolymerization and repolymerization studies of PET

**DOI:** 10.1039/d3ra07376a

**Published:** 2023-12-13

**Authors:** Yang Yu, Guoliang Shen, Tie Jun Xu, Ruiyang Wen, Yun Chang Qiao, Ru Chao Cheng, Yue Huo

**Affiliations:** a Shenyang University of Technology Liaoyang City Liaoning Province China shengl_shxy@sut.edu.cn

## Abstract

In this study, a Ti–Si–ethylene glycol salt (Ti/Si–EG) was synthesized and used as a catalyst for the depolymerization of PET–ethylene glycol to produce bis(hydroxyethyl)terephthalate (BHET), and the optimum conditions for the depolymerization were determined by response surface analysis: *w*(EG) : *w*(PET) was 4 : 1, the catalyst mass dosage was 0.56% (in terms of the mass of PET), the depolymerization temperature was 203 °C, the depolymerization time was 3.8 h, and the highest yield of the product (BHET) was 90.10%. Furthermore, the depolymerization product BHET was used as the raw material and Ti/Si–EG was used as the catalyst for the polycondensation reaction to synthesize PET, and the amount of the catalyst added was 0.015% by mass (in terms of BHET). The performance of the synthesized PET was tested to be the same as that of PET synthesized by the PTA method under the same conditions, thus achieving the resourceful treatment and high-value recycling of waste PET. The introduction of Si in the catalyst reduced the catalytic activity of Ti and avoided the problem of yellowing of polyester products, and the use of the glycol salt as a catalyst in the depolymerization and polycondensation reaction process did not introduce impurity groups.

## Abstract 2

In this study, a Ti–Si–ethylene glycol salt (Ti/Si–EG) was synthesized and used as a catalyst for the depolymerization of PET–ethylene glycol to form bis(hydroxyethyl)terephthalate (BHET), and catalysts for the resynthesis of PET by BHET.

## Graphical abstract

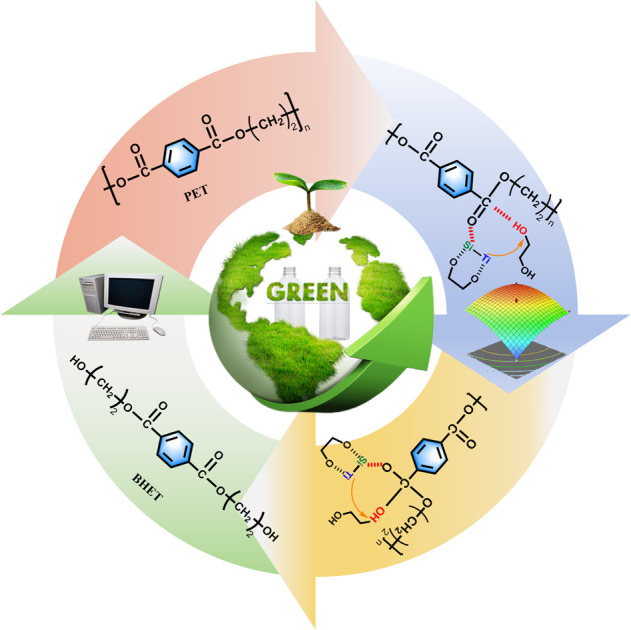

## 1. Introduction

Polyethylene terephthalate (PET) is widely used in various aspects of life because it is non-toxic, odorless, transparent, stable, and easy to process. Currently, the global production of PET continues to rise, but the recycling rate is less than 10%.^1^ The good stability of PET makes it difficult to naturally depolymerize; therefore, the development of efficient PET recycling technology is of great significance to alleviate the pressure on the environment.^2,3^

Currently, waste PET recycling and reuse methods are divided into three categories: chemical recycling^4–9^ which is highly efficient in terms of the reagents used to depolymerize PET and is an effective way to realize the sustainable use of plastic recycling; physical recycling, which uses high-temperature melt extrusion to recycle PET for a second time, but the quality index of the recycled PET will be reduced, limiting the number of recycling times and compromising the quality of the recycled product; and biological recycling,^10–13^ which is similar to chemical recycling and depolymerizes PET, but it is more costly. At present, the use of ethylene glycol as a solvent has been reported to depolymerize PET, and the yield of the depolymerization product BHET is mostly in the range of 50–80% and seldom exceeds 90%.^14^ In this regard, it is necessary to develop a suitable depolymerization catalyst.

Re-polymerization of the depolymerization product BHET can undoubtedly achieve the closed-loop utilization of PTE. At present, more than 90% of the catalysts used in the industrial synthesis of PET are antimony-based, but they are potentially toxic to the environment and organisms; therefore, more and more studies are being focused on titanium-based catalysts,^15,16^ which have a high catalytic activity and are environment friendly and inexpensive. However, studies have shown that titanium catalysts have certain drawbacks, such as a very high catalytic activity and too many catalytic side reactions in the process, which limits their large-scale application.

In order to recycle PET, it is required that the catalyst meets the following conditions: (1) the catalyst is safe and has no direct or potential toxicity toward human beings and organisms. (2) The catalytic activity should not be too high, so as to avoid problems such as deepening of the color of the product and side reactions caused by the catalytic activity during the depolymerization and polycondensation processes. (3) No impurity groups should be introduced into the depolymerization and polycondensation system to avoid the introduction of impurity groups causing the quality of BHET to be lowered, as well as the introduction of the impurities in the process of polycondensation, resulting in the PET product being prematurely capped, the molecular weight of the product difficult to increase and other problems.^17–21^

In this study, Si, which has a huge stock in nature, is non-toxic, non-hazardous, cheap, and easily available, was chosen to be used in conjunction with Ti.^22–27^ The ethylene glycol salt of Ti–Si (Ti/Si–EG) was prepared for use as a catalyst in this study. Because the arrangement of the extra-nuclear electron cloud of the element silicon (Si) makes it weakly catalytic when used alone, its use in combination with the element titanium (Ti) can reduce the activity of the titanium catalyst.^28^ As a glycol salt, the catalyst will not introduce impurity groups during depolymerization and polycondensation, thus affecting the quality of the product. Moreover, after Ti/Si–EG is catalyzed, the deactivated catalyst is uniformly dispersed in PET and can be used as a matting agent.^29^

## 2. Materials and methods

### 2.1 Materials and instruments

#### 2.1.1 Materials

Waste PET, tetrabutyl titanate, tetraethyl orthosilicate, xylene, anhydrous ethanol, terephthalic acid (PTA), ethylene glycol (EG), and the raw materials used in the experiments were all analytically pure reagents.

#### 2.1.2 Instruments

A balance (FA2204B), China Shanghai Precision Scientific Instruments, viscometer, China, field emission scanning electron microscope (SEM, Apreo2C) Thermo Fisher Scientific, U.S.A., infrared spectrometer (TENROR-II) Bruker, Germany, thermogravimetric analyzer (TG, STA 449 F3), Bruker, Germany, X-ray diffraction spectrometer (XRD, D8 Advance) Bruker, Germany, differential scanning calorimeter (DSC, DSC822e) METTLER TOLEDO, Switzerland, colorimeter (ADCI-60-C) China, circulating water vacuum pump (SHZ-D), China, laser particle size distribution meter (BT-800), China, gel permeation chromatography (GPC, 1260) Agilent, U.S.A.

### 2.2 Methods

#### 2.2.1 Synthesis of Ti/Si–EG catalyst

**Fig. 1:**
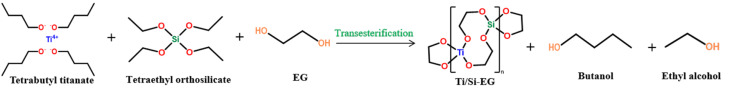
Preparation of Ti/Si–EG catalysts.

Tetrabutyl titanate, tetraethyl orthosilicate, and ethylene glycol were mixed in a 1 : 1 : 4 (mol) ratio and added to the reactor. The mixture was then heated to 115 °C and refluxed for 3 h. Xylene was added as a solvent. The reaction mechanism was changed from reflux to distillation. Cooling was performed once the desired amount of the product was obtained. The solid product was washed with anhydrous ethanol, dried, and pulverized to produce the Ti–Si–EG catalyst (Fig. 1).

#### 2.2.2 Depolymerization procedures

Waste PET was washed, dried, and added to the reactor. Ethylene glycol was added, and the temperature was raised to approximately 100 °C. The Ti/Si–EG catalyst was added, and the mixture was refluxed at around 200 °C for 3–4 hours. After the reaction was complete, the temperature was reduced, and distilled water was added to the reaction system. When the temperature reached 60 °C, the mixture was filtered, to separate the incompletely depolymerized polymers, and the filtrate was cooled at 5 °C for 5 hours. The resulting crystallized BHET product was filtered, vacuum-dried, and obtained as the final product.

#### 2.2.3 Synthesis of PET

**Fig. 2:**
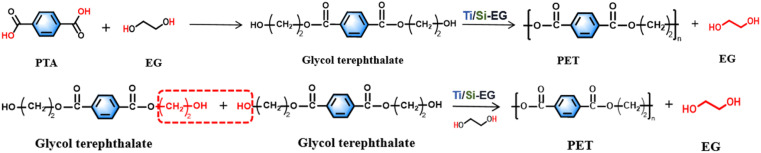
Synthesis of PET.

The synthesis steps for PET are shown in Fig. 2.

(1) The synthesis of PET by the PTA method begins with an esterification stage, wherein terephthalic acid and ethylene glycol undergo an esterification reaction to produce ethylene glycol terephthalate (*x* = 1).

(2) Glycol terephthalate removes the hydroxyl group (–OH) and the hydroxyethyl group (–CH_2_CH_2_OH) from each end in the beginning stage, and the two are combined to regenerate EG. Subsequently, the monomers with the groups are removed from both ends and are connected to each other to form polyethylene terephthalate with a low degree of polymerization (*x* = 1–4 or so), which is the pre-condensation stage.

(3) The polycondensation stage repeats the chain-enhancing reaction on the basis of (2) at reduced pressure and elevated temperature to generate large molecular weight PET.

The synthesis of PET using the BHET method omits the esterification stage and proceeds directly to the pre-condensation and condensation steps of (2) and (3), as described above. The specific method is as follows the raw material terephthalic acid (PTA) and ethylene glycol were added to the reactor, and the total amount of the catalyst added was 0.015% of the raw material amount, and the catalyst was uniformly dispersed in ethylene glycol before use. The esterification reaction was carried out under the conditions of atmospheric pressure esterification, and temperature between 185–195 °C, and a stirring speed of about 100 rpm. The polycondensation test was carried out between 260–280 °C, stirring speed of 100 rpm, and pressure of −0.1 MPa.

#### 2.2.4 Testing of molecular weight

##### Viscosity tests

The molecular weight parameters presented herein were obtained from a solvent mixture of phenol : tetrachloroethane of 1 : 1 (w%), tested in a constant temperature water tank at 25 °C using a Ubbelohde viscometer.1
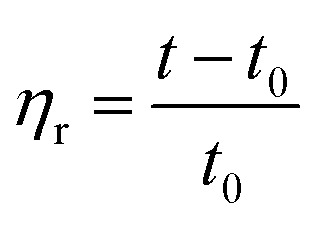
2
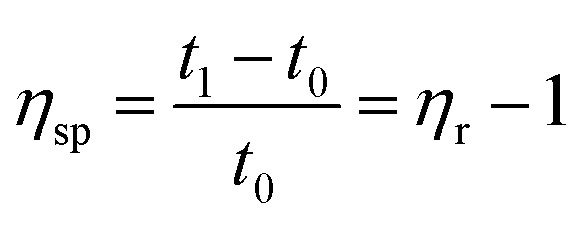
3
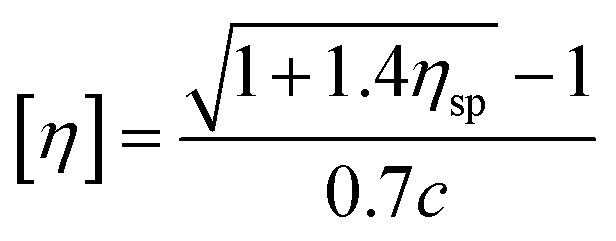


According to eqn (1)–(3), *η*_r_ is the relative viscosity, *t*_1_ is the solution flow time through the capillary (s), *t*_0_ is the solvent flow time through the capillary (s), *η*_sp_ is the incremental viscosity, [*η*] is the characteristic viscosity (dL g^−1^), and *c* is the solution concentration (g dL^−1^). The average molecular weight of PET was deduced from the measured characteristic viscosity.

## 3. Results and discussion

### 3.1 Characterization of Ti/Si–EG

#### 3.1.1 Topography analysis

**Fig. 3:**
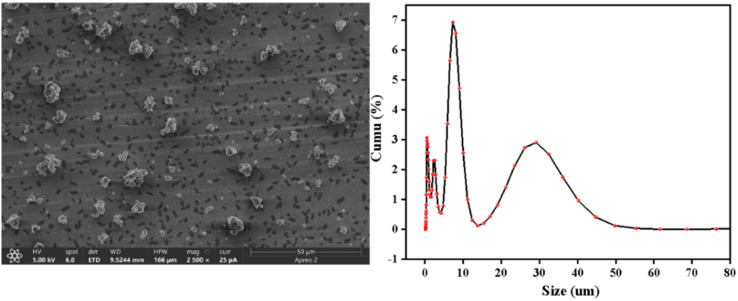
The SEM image and size of the catalyst.

The scanning electron microscope test results of the Ti/Si–EG catalyst are shown in Fig. 3. It can be seen that the Ti/Si–EG has a cross-linked flake structure, and the particle size distribution is in the range of 0–50 μm. After testing, Ti/Si–EG had good dispersibility in ethylene glycol. It can be stably dispersed in ethylene glycol for more than 30 min, which meets the requirements for use.

#### 3.1.2 XRD analysis

**Fig. 4:**
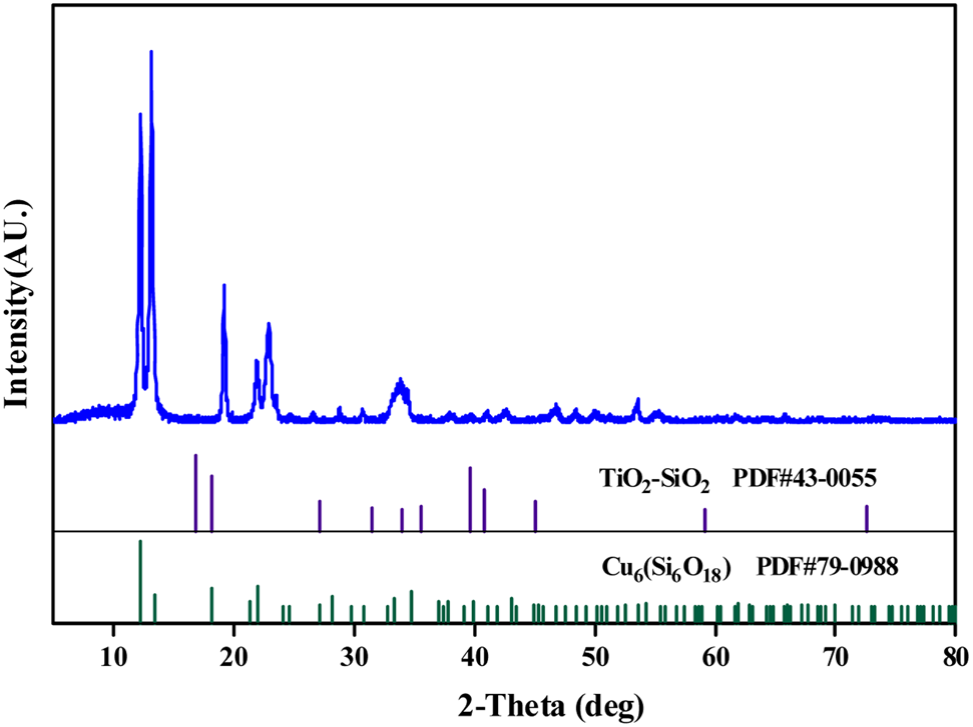
XRD pattern of the catalyst.

Fig. 4 shows an XRD pattern of the catalyst, it can be seen that Ti/Si–EG exists with crystalline features of TiO_2_–SiO_2_ (PDF #43-0055) and Cu_6_(Si_6_O_18_) (PDF #79-0988).

#### 3.1.3 IR analysis

**Fig. 5:**
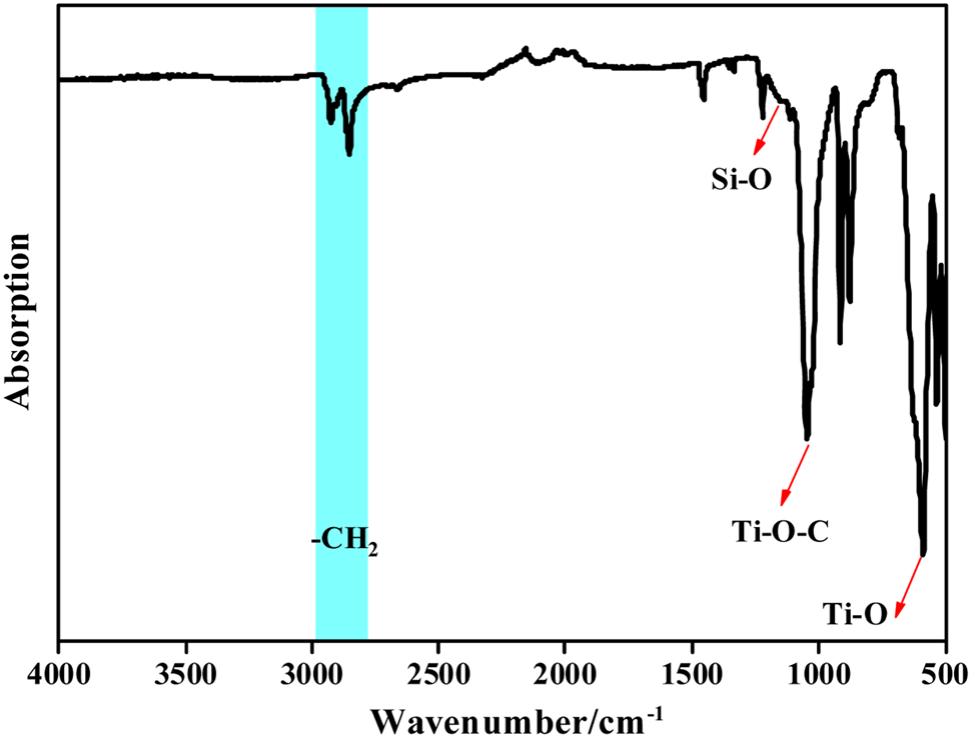
FT-IR of the Ti/Si–EG catalyst.

Fig. 5 shows FT-IR spectra of Ti/Si–EG, in which the peaks at 2798.1–2953.7 cm^−1^ correspond to the stretching vibration of –CH_2_, the strong absorption peak at 1045.9 cm^−1^ corresponds to the stretching vibration of the Ti–O–C bond, and the absorption peak at 587.9 cm^−1^ corresponds to the stretching vibration of Ti–O. The absorption peak at 1150.1 cm^−1^ corresponds to the vibration of the Si–O bond.

#### 3.1.4 TG analysis

**Fig. 6:**
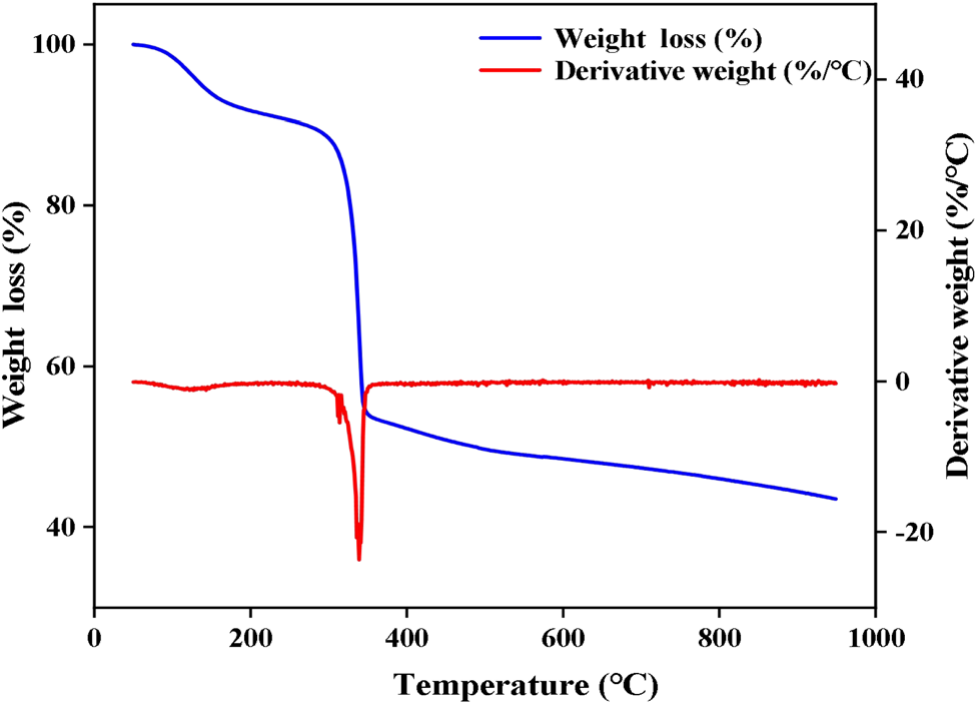
TG curve of the catalyst.

TG curves of Ti/Si–EG are shown in Fig. 6, in which the first weight loss section before 150 °C corresponds to desolventization. After 150 °C, with the adsorbed solvent coming off, Ti/Si–EG loses weight for the second time near 350 °C. Therefore, Ti/Si–EG meets the required conditions for polyester catalysis after desolventization. Before use, it must be dried at a constant temperature of 150 °C.

### 3.2 Characterization of BHET

#### 3.2.1 DSC analysis

**Fig. 7:**
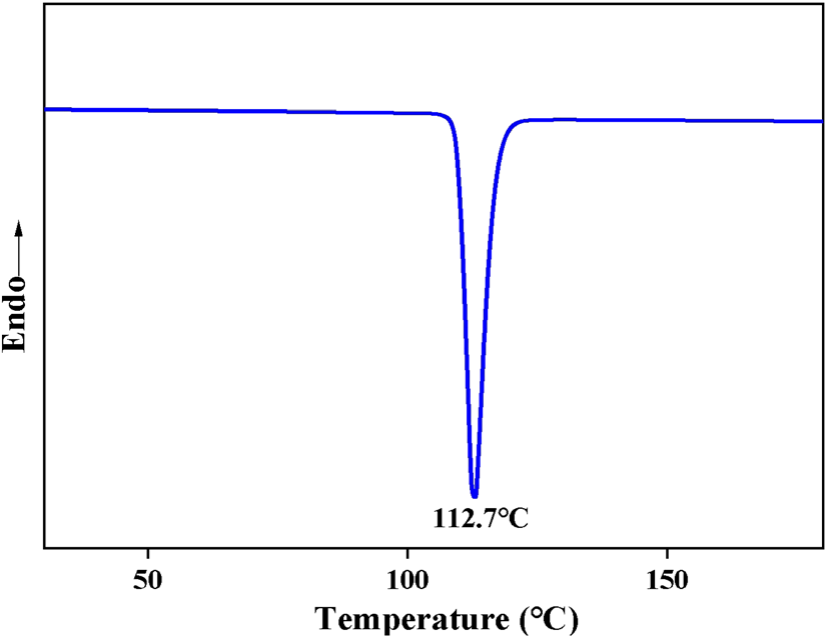
DSC curve of the depolymerization product BHET.

Fig. 7 shows the DSC temperature increase curve of the PET depolymerization product, where the temperature was increased from 25 °C to 200 °C in an N_2_ atmosphere at a rate of 15°C min^−1^. It can be seen that the melting point of the BHET monomer is approximately 112.7 °C, and therefore DSC testing of the depolymerization product can be used to initially determine the presence of BHET.

#### 3.2.2 IR analysis

**Fig. 8:**
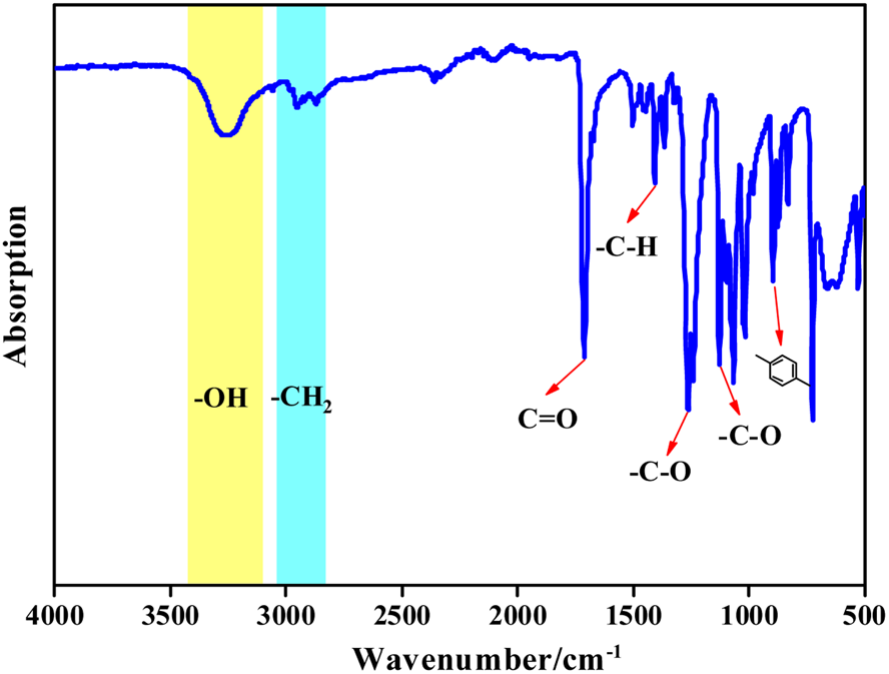
FT-IR spectra of the depolymerization products.

From Fig. 8, it can be seen that the absorption peak at 3257.7 cm^−1^ corresponds to the stretching vibration of the BHET terminal hydroxy –O–H, the strong absorption peak at 1709.5 cm^−1^ corresponds to the stretching vibration of the ester C=O, vibration peak of the aromatic –C–H at 1405.3 cm^−1^, C–O vibration peaks at 1261.4 cm^−1^ and 1125.8 cm^−1^, and the bending vibration in the aromatic ring at 896.1 cm^−1^. Vibration peaks at 2952.2 cm^−1^ and 2868.2 cm^−1^ are due to –C–H of the methylene in the EG chain. The infrared spectrum of the product is consistent with the infrared spectrum of BHET, which confirmed that the copolymer product was BHET.

#### 3.2.3 ^1^H NMR analysis

**Fig. 9:**
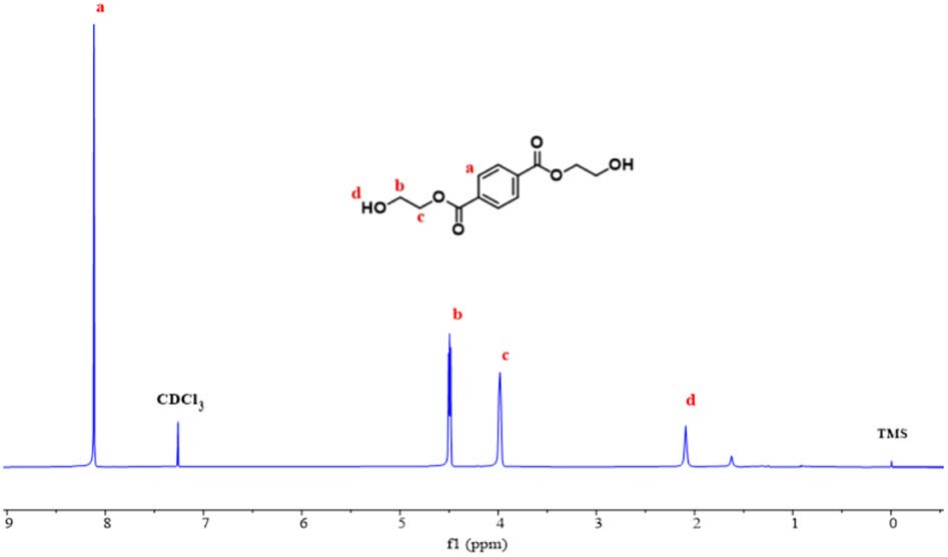
^1^H NMR of depolymerization products.

^1^H NMR spectrum of the depolymerization product is shown in Fig. 9, ^1^H NMR (400 MHz, CDCl_3_) *δ* 8.12 (s, 4H), 4.52–4.43 (m, 4H), 4.07–3.98 (m, 4H), 2.09 (s, 2H). Therefore, it can be determined that the product is BHET.

### 3.3 Characterization of PET

#### 3.3.1 IR analysis

**Fig. 10:**
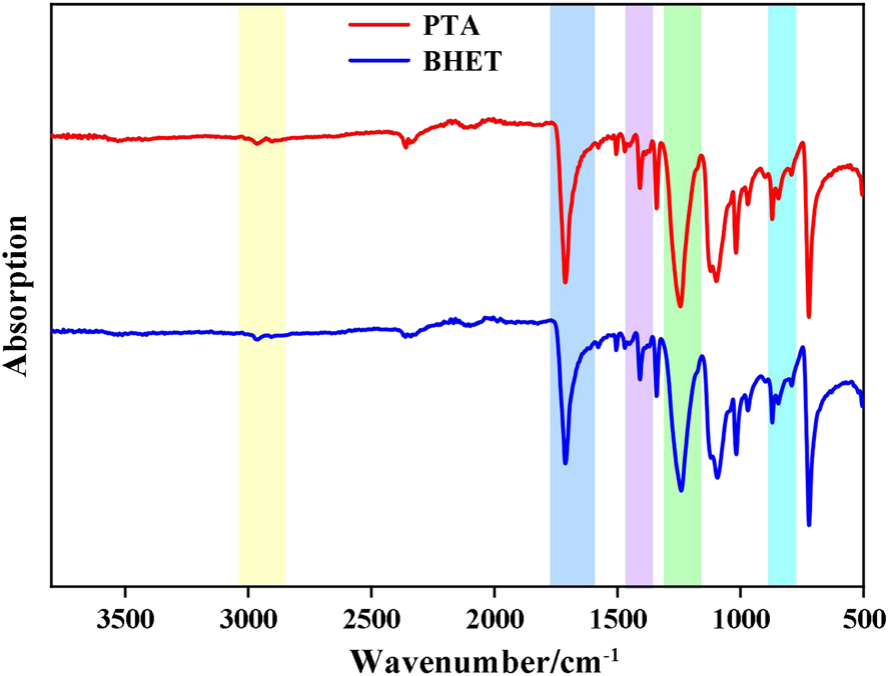
FT-IR spectra of PET and BHET.

The FT-IR spectrum of the synthesized PET is shown in Fig. 10. The strong absorption peaks at 1650–1770 cm^−1^ correspond to the telescopic vibration of ester C=O, aromatic –C–H vibration peaks at 1390–1450 cm^−1^, C–O vibration peaks at 1160–1300 cm^−1^ and 1050–1150 cm^−1^, and –C–O vibration peaks at 850–890 cm^−1^. The peaks at 1160–1300 cm^−1^, 1050–1150 cm^−1^, and 850–890 cm^−1^ correspond to aromatic ring bending vibrations. The C–H vibration of the methylene group in the EG chain was observed at 2850–2980 cm^−1^. The infrared spectra of the catalyst-synthesized products were consistent with those of PET, which confirmed that the catalytic products were all PET.

#### 3.3.2 DSC analysis

**Fig. 11:**
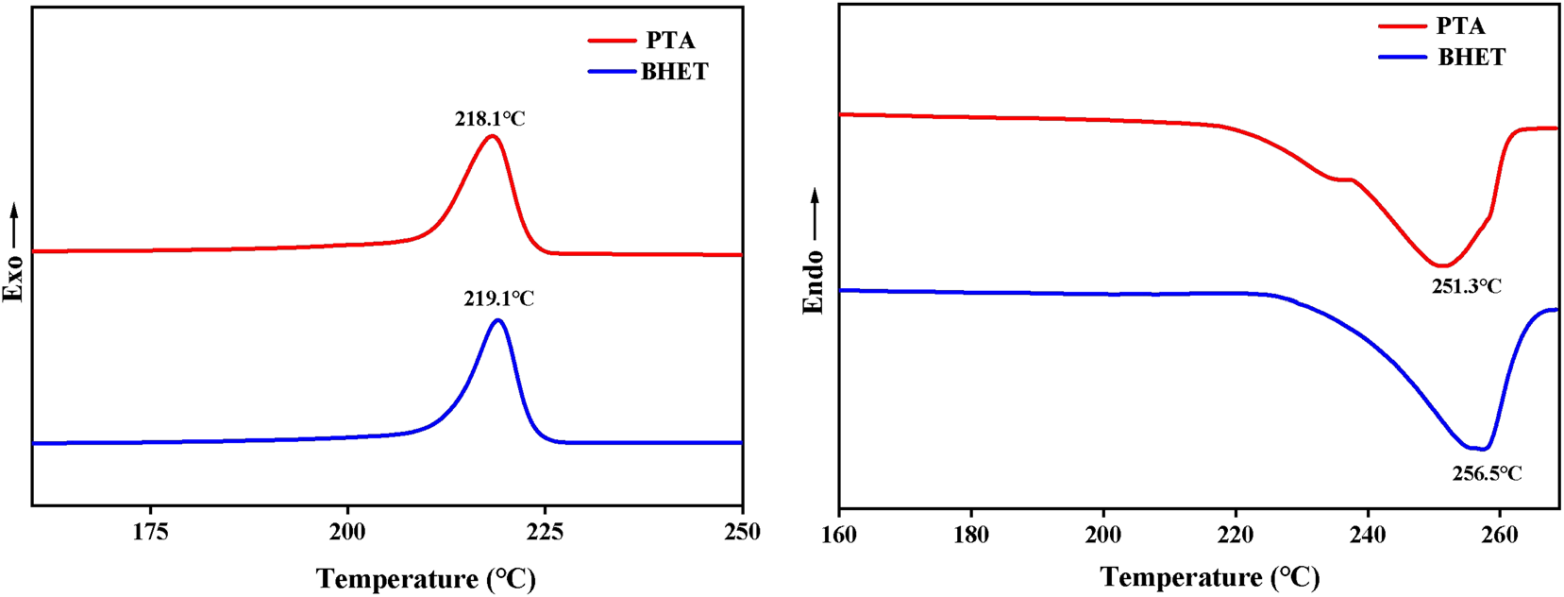
DSC curves of PET and BHET.

The nitrogen flow rate was 40 mL min^−1^, and the program temperature was 10°C min^−1^ from −10 °C to 280 °C, holding for 10 min, and then 10°C min^−1^ to −10 °C, holding for 10 min for the DSC analysis. As shown in Fig. 11 for the DSC test results of the synthesized PET, it can be seen that there was not much difference in the melting temperature and crystallization temperature between the PET re-synthesized using the depolymerization product BHET and the PET synthesized by the traditional PTA method. The thermal properties of the two are basically the same.

#### 3.3.3 GPC analysis

**Fig. 12:**
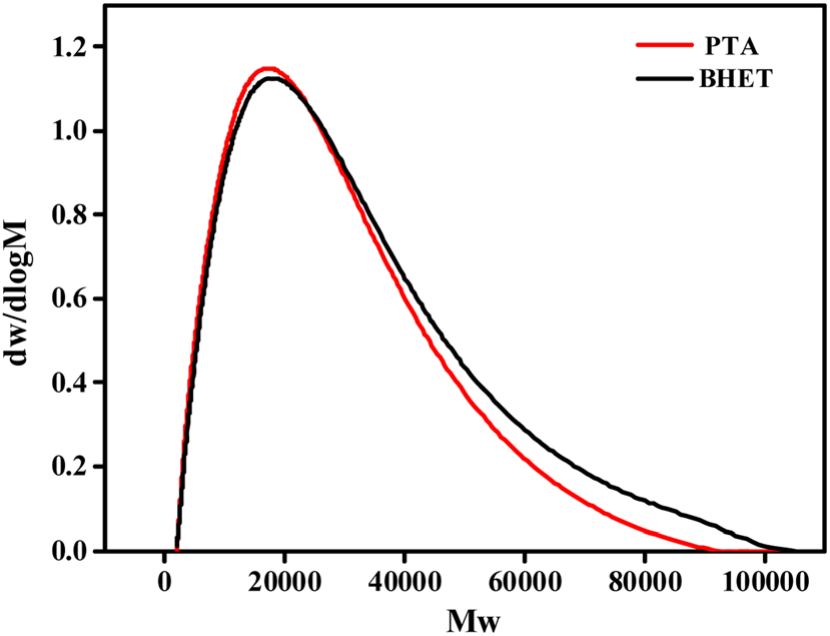
GPC outflow curves for synthetic PET.

The GPC effluent profile of the synthesized PET is shown in Fig. 12. The results of the gel permeation chromatography analysis of the synthesized PET using THF as a solvent showed that the molecular weight distribution peaks of the PET synthesized by the two methods are similar. PET synthesized by the PTA method has a narrower range of molecular weight distribution peaks and smaller PDI values, which also predicts a more uniform molecular weight distribution. However, the difference between the PET synthesized by the two methods is small. The GPC measurements of the synthetic PET are shown in Table 1.

**Table 1:** GPC measurement results of synthetic PET^a^

Name	*M*_n_	*M*_w_	PDI
PTA	11 048	17 581	1.591
BHET	11 521	18 484	1.604

a *M*_n_ is the number average molecular weight; *M*_w_ is the weight average molecular weight; and PDI = *M*_w_/*M*_n_, which represents the molecular weight distribution index.

### 3.4 Response surface experiment results

Based on the Box–Behnken model,^30^ experiments were designed. Response surface modeling was performed using software for the analysis. The experimental results of the corresponding results of the response surface curves are as follows.

**Fig. 13:**
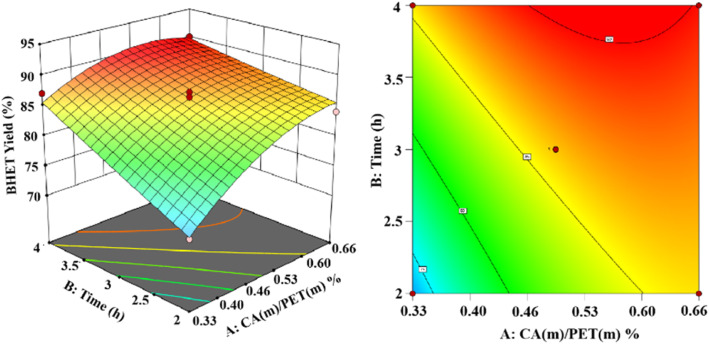
The effect of the catalyst dosage and reaction time on BHET yields.

The 3D and 2D response surface plots created to show the effect of the interaction between the catalyst use and reaction time on the yield of BHET are shown in Fig. 13 for a fixed reaction temperature of 200 °C. The yield of BHET increased with the reaction time and gradually leveled off beyond four hours. However, the polymerization of ethylene glycol occurred with the extension of the reaction time, which led to the deepening of the color of the obtained BHET. The yield of BHET increased with the increase of the catalyst dosage, and it tended to stay constant when the catalyst mass dosage was greater than 0.6% (in terms of the PET mass).

**Fig. 14:**
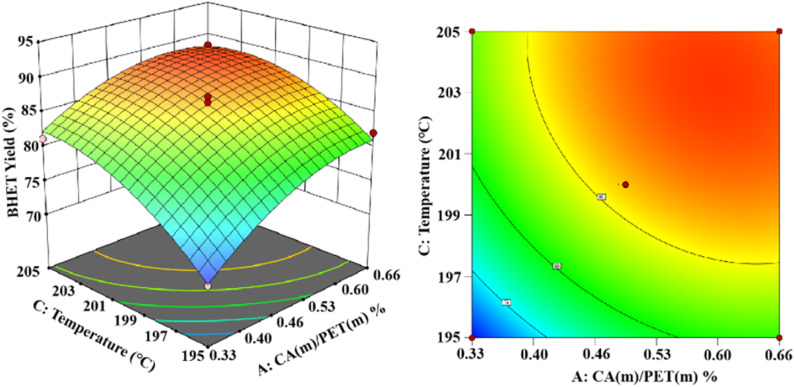
The effect of catalyst dosage and reaction temperature on BHET yields.

As shown in Fig. 14, the established 3D and 2D response surface plots showed the effect of the interaction between the catalyst use and the reaction temperature on the BHET yield. Fixing the reaction time to 3 h, the BHET yield gradually increased with the increase in the reaction temperature. However, when the reaction temperature exceeded the boiling point of ethylene glycol, the reflux of the reaction system was intensified, and the yield of the product BHET tended to be constant. However, the high temperature also intensified the polymerization of ethylene glycol, making the color of the product BHET deepen. The yield of BHET increased with the catalyst dosage and tended to be constant when the catalyst mass dosage was greater than 0.6% (in terms of the PET mass).

**Fig. 15:**
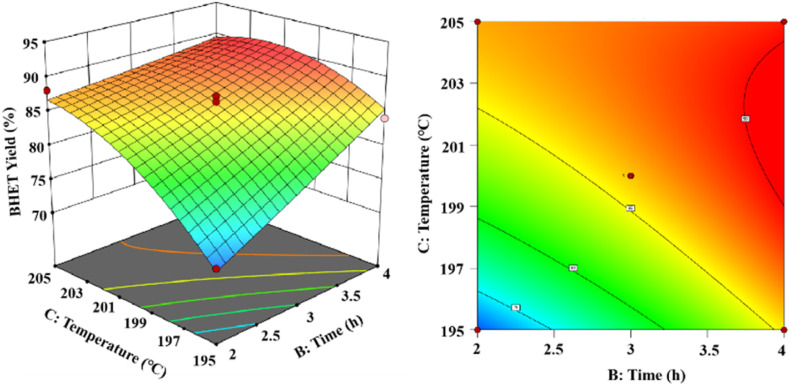
The effect of reaction time and reaction temperature on the yield of BHET.

As shown in Fig. 15, the 3D and 2D response surface plots were created to show the effect of the interaction between reaction time and reaction temperature on the BHET yield. The amount of the fixed catalyst mass was 0.6% (in PET mass). The BHET yield gradually increased with the increasing reaction time and reaction temperature. As the temperature increases the yield of BHET tends to be constant at the boiling point of ethylene glycol, and prolongation of the reaction time revealed that the BHET yield tended to be constant after 3.5 h of reaction time.

Ultimately, the variance of the regression equation showed that the yield of BHET increased upon increasing the reaction temperature, reaction time, and catalyst dosage. The interactions were significant between catalyst dosage and reaction time, less significant between the catalyst dosage and reaction temperature, and more significant between the reaction time and reaction temperature. The optimal depolymerization conditions were determined to be a reaction temperature of 203 °C, reaction time of 3.8 h, and maximum yield of 90.1% for BHET when the catalyst mass dosage was 0.56% (in terms of the PET mass).

**Fig. 16:**
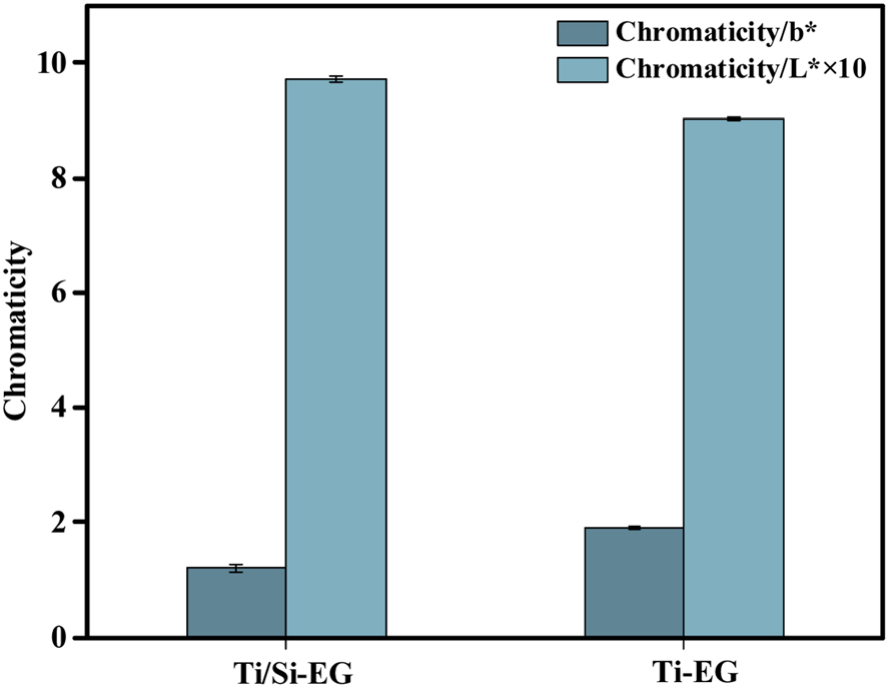
Chromaticity of depolymerization products.

As shown in Fig. 16, the BHET monomer depolymerized using Ti/Si–EG as a catalyst under the optimal process conditions had a b* of 1.2 and an L* of 97.1, and the BHET monomer was depolymerized using Ti–EG catalyst under the same conditions was significantly darker than the BHET depolymerized using Ti/Si–EG alone. This phenomenon is related to the excessive catalytic activity and poor catalytic selectivity of Ti–EG.

### 3.5 Molecular weight and chromaticity analysis of synthetic PET

**Fig. 17:**
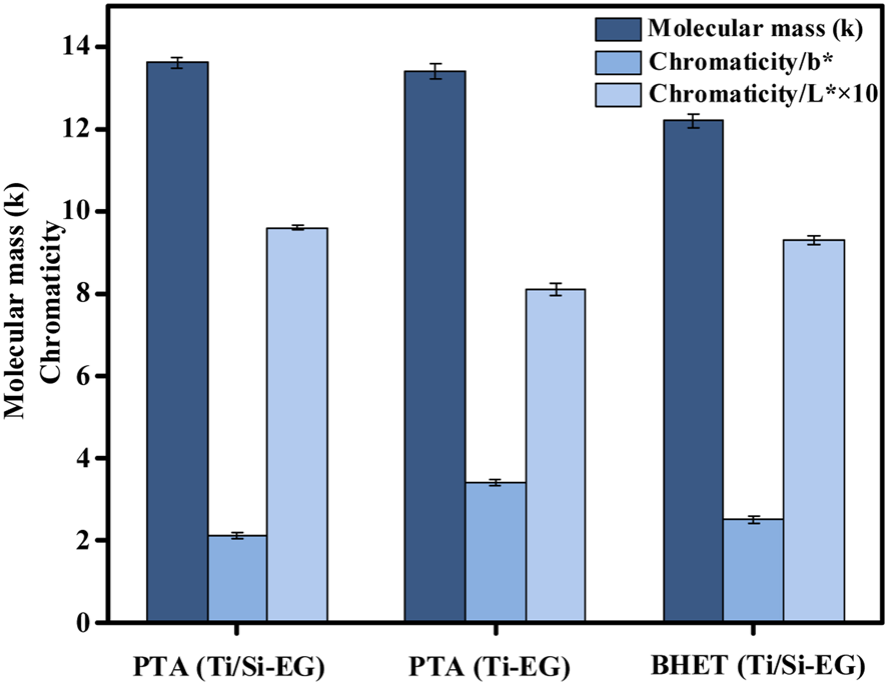
Molecular weight and chromaticity of synthetic PET.

As can be seen in Fig. 17, the color (L*, b*) and molecular weight indexes of PET under the above PET synthesis conditions were examined under different cases: (1) PET synthesized using PTA as the raw material and Ti/Si–EG as the catalyst had the lowest b*, the highest L*, and the lightest overall color. (2) PET synthesized using PTA as the raw material and Ti–EG as the catalyst had the highest b*, lowest L*, and darkest color. (3) PET synthesized using BHET as the feedstock and Ti/Si–EG as the catalyst had in between b* and L* values.

The reason for this phenomenon is the high catalytic activity and poor selectivity of the single Ti–EG catalyst at high temperatures. The catalyst catalyzes the polymerization of ethylene glycol resulting in the darkening of the PET color. Under the joint action of Ti and Si elements, it can be seen from the above data that the activity of Ti was suppressed, and the color of the product was reduced.

PET synthesized from the depolymerization product BHET using Ti/Si–EG as the catalyst showed a small but insignificant increase in chromaticity compared with that synthesized by the PTA method. The reasons may include polymerization of the glycol during depolymerization, excessive depolymerization of PET, and oxidation of some additives in commercial PET. However, the color deepening of BHET is not obvious and meets the index of reuse.

### 3.6 Depolymerization and condensation mechanisms of Ti/Si–EG catalysts

**Fig. 18:**
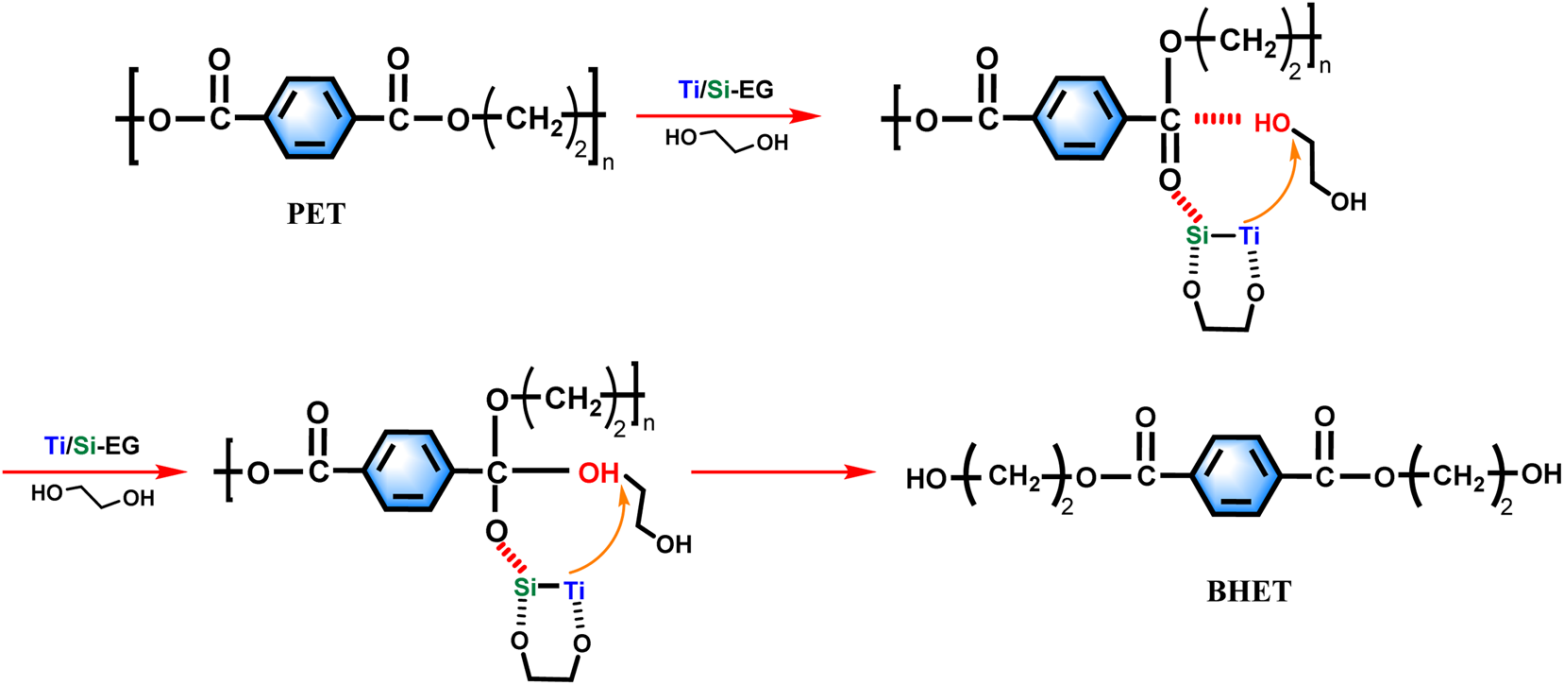
Reaction mechanism.

Based on the experimental results of this study, the experimental mechanism of ethylene glycol depolymerization of waste PET using Ti/Si–EG as a catalyst is shown in Fig. 18.

The depolymerization process was carried out in two steps. The first step was the activation process of the catalyst, in which Ti, Si, and O in Ti/Si–EG formed a coordination structure with the hydroxyl groups of ethylene glycol, which increased the electronegativity of the hydroxyl oxygen in EG. Ti and Si cations interacted with the oxygen in the PET ester group, which makes it easier for the hydroxyl oxygen on EG to attack the positively charged carbon ions in the PET ester group and nucleophilic substitution occurs to form tetrahedral structural intermediates. Subsequently, the H on EG is detached, the C=O bond on the PET ester group is broken, and the electrons on the other oxygen of the PET ester group are transferred to form a new –C–O bond. As –OCH_2_CH_2_– is detached from PET, the PET chain is broken. The above process is repeated until the monomer is formed.

During the polycondensation reaction of PET, the catalyst ligand first undergoes an exchange reaction between the end hydroxyl groups of the monomer or oligomer to form a metal alcohol salt. The oxygen in –OCH_2_CH_2_– in the other molecule attacks the carbonyl carbon atom, and the nucleophilic attack of the ester carbonyl oxygen on the central titanium atom increases the positive charge of the carbonyl carbon, thereby increasing the rate of reaction.

## 4. Conclusions

In this study, Ti–Si–ethylene glycol salt (Ti–Si–EG) was synthesized by transesterification of titanate and silicate with ethylene glycol, combining Si, of low polyester catalytic activity, with Ti, of high catalytic activity. This solved the problem of high activity when using a single Ti catalyst in PET catalysis. Also, the catalyst as a glycol salt avoids the problem of introducing impurity groups during PET synthesis and depolymerization.

Glycol depolymerization of PET was carried out using the synthesized catalysts. Response surface methodology was utilized to determine the optimum process conditions, which included a *w*(EG) : *w*(PET) ratio of 4 : 1, a catalyst dosage of 0.56% (based on the mass of PET), a reaction temperature of 203 °C, and a depolymerization time of 3.8 hours. As a result, the yield of the product (BHET) could reach 90.10%.

The same catalyst was used again for the polycondensation of the depolymerized product BHET, and the obtained product PET was tested for color, molecular weight, and thermal properties. It can be concluded that PET synthesized by this method is the same as the PET synthesized by the PTA method in terms of various indexes. Compared with a single titanium catalyst, the titanium–silicon composite catalyst can significantly reduce the chromaticity b* of the depolymerization product and the condensation product. This study provides a reference for the resource recovery of waste PET through the synthesis of catalysts.

## Author contributions

Prof. Guoliang Shen and Prof. Tie Jun Xu provided the idea for the experiment, Yang Yu led the experimental work, Wen Ruiyang completed the data organization, Yue Huo provided the data analysis and other aspects of the work, Yun Chang Qiao and Ru Chao Cheng assisted to complete the work for sample testing.

## Conflicts of interest

The authors declare that they have no conflicts of interest.

## Supplementary Material
